# Screen time and attention-deficit/hyperactivity disorder in children: a systematic review and meta-analysis

**DOI:** 10.3389/fped.2026.1860549

**Published:** 2026-09-28

**Authors:** Caihong Zhao, Jing Xu, Xirui Wu, Yufen Li

**Affiliations:** 1 Department of Child Health Care, Qingdao Maternal and Child Health and Family Planning Service Center, Qingdao, China; 2 Department of Neurology, Qingdao Central Hospital, University of Health and Rehabilitation Sciences, Qingdao, China; 3 Quality Control Department, Qingdao Central Hospital, University of Health and Rehabilitation Sciences, Qingdao, China

**Keywords:** attention-deficit/hyperactivity disorder (ADHD), children, meta-analysis, neurodevelopment, preschoolers, screen time

## Abstract

**Background:**

Children's exposure to electronic screens has risen sharply. This study quantitatively evaluated the association between excessive screen time and childhood attention-deficit/hyperactivity disorder (ADHD) risk through a systematic review and meta-analysis, exploring the moderating roles of age and media type.

**Methods:**

Following PRISMA guidelines, we systematically searched observational studies assessing children's screen time and ADHD, and finally included 10 observational studies with overall low-to-moderate risk of bias. Risk of bias was evaluated using the ROBINS-I tool. Continuous data were unified using the Chinn conversion rule. A DerSimonian-Laird random-effects model calculated pooled odds ratios (OR) and 95% confidence intervals (CI). Subgroup analyses, leave-one-out sensitivity analyses, and Begg's and Egger's tests were also conducted.

**Results:**

Excessive screen time was significantly associated with elevated childhood ADHD risk (pooled OR = 1.56, 95% CI: 1.31–1.87, *P* < 0.001). Substantial between-study heterogeneity was observed (Q = 344.51, I^2^ = 97.4%, *P* < 0.001). Subgroup analyses revealed significant age-related heterogeneity: preschoolers (0–6 years) showed a larger magnitude of association (OR = 2.40, 95% CI: 1.44–3.98), whereas the association in school-age children and adolescents (>6 years) was of smaller magnitude (OR = 1.16, 95% CI: 1.05–1.29). Passive TV/video viewing showed a higher point estimate of ADHD risk (OR = 2.20) than interactive digital media (OR = 1.10), but the between-group difference did not reach statistical significance. Sensitivity analyses confirmed the robustness of these findings.

**Conclusions:**

Excessive screen exposure is significantly associated with elevated ADHD risk in children, with distinct age-specific and media type-specific patterns. The preschool period appears to be a high-risk window for screen-related neurodevelopmental effects, with a trend toward stronger association for passive screen exposure. Public health and clinical interventions should prioritize restricting early screen exposure, particularly passive, fast-paced videos without substantial interaction.

## 1 Introduction

Attention-deficit/hyperactivity disorder (ADHD) is one of the most common neurodevelopmental disorders in childhood, with a global prevalence of approximately 5.9%–7.2% (1). Its core symptoms include inattention, hyperactivity and impulsivity, which not only seriously affect children's academic performance, social function and family interactions, but may also persist into adulthood, leading to occupational maladjustment, psychological disorders and long-term personal and social burdens (2).

With the rapid popularization of digital technology, electronic screens such as televisions, smartphones, tablets and computers have been deeply integrated into children's daily lives, becoming important carriers for their entertainment and learning. The World Health Organization (WHO) released guidelines in 2019 that clearly recommend no screen exposure for children under 2 years old, and a daily screen time of no more than 1 h for children aged 3–5 years (3). However, latest global epidemiological data indicate that real-world screen use widely deviates from these recommendations: approximately 65% of children aged 3–5 years exceed the 1-hour daily limit, with an average daily duration of 2.1 h, and screen exposure rates among children under 2 years reach up to 70% in some regional cohorts (3, 4). This makes children's screen use a pressing global public health concern.

The impact of screen exposure on children's cognitive and neurodevelopment has become a hot research topic in academia, and several widely recognized hypotheses have been formed. The dopamine reward system hypothesis holds that fast-paced, high-sensory-stimulus screen content can overactivate the brain's dopamine reward pathway, reducing children's concentration and tolerance for low-stimulation tasks in real life, such as reading, classroom learning and face-to-face communication (5). The displacement hypothesis points out that excessive screen time will take up the time that children should spend on outdoor activities, adequate sleep, high-quality parent-child interaction and physical play—these activities are crucial for the development of the prefrontal cortex and the construction of executive functions (6). In addition, disruption of sleep rhythms by screen blue light and inhibition of active attention by passive screen viewing have also been proposed as potential pathways through which screen exposure may influence neurodevelopment.

Although a large number of studies have explored the association between screen time and ADHD risk, the existing evidence is still controversial and insufficient. Some studies have observed that excessive screen exposure is a significant predictor of ADHD symptoms in children even after adjustment for major confounding factors (7), while others suggest that this association may be interfered by confounding factors such as family socioeconomic status, parenting style and genetic susceptibility (8, 9). There is even a view of “reverse causality"—children with hyperactive tendencies are more likely to be attracted by high-stimulation screen content to seek sensory balance (8). At the same time, most existing studies do not distinguish between different types of screen media; passive TV viewing and interactive digital media such as educational games and tablet learning applications may have essentially different effects on the nervous system. Moreover, the plasticity of brain development varies greatly in different age stages, and the vulnerability of children at different developmental stages to screen exposure has not been systematically quantified.

Prior meta-analyses on this topic have advanced understanding but also have notable limitations that leave important evidence gaps. Early meta-analyses, such as the work by Christakis et al. and Zimmerman & Christakis (5, 10), focused predominantly on a single traditional media type (television) and were limited to preschool populations, making them unable to reflect the current landscape of multi-device digital exposure. A subsequent meta-analysis by Beyens et al. (6) synthesized studies across four decades and covered a wide age range from infancy to adolescence, but did not conduct stratified analyses by age group or media type, and thus could not quantify potential age-specific vulnerabilities or differential effects of passive versus interactive screen content. To date, no systematic review and meta-analysis has simultaneously examined the moderating roles of both age and media type on the association between screen time and ADHD, leaving key questions about high-risk populations and high-risk exposure patterns unresolved. In addition, several large-scale population-based studies have been published in recent years, including national survey data and prospective cohort studies, which provide an opportunity to update and extend earlier evidence with larger samples and more diverse exposure types. From a developmental perspective, preschool-aged children are hypothesized to be particularly vulnerable to screen-related effects due to the rapid maturation of prefrontal circuits during this period, while evidence for school-age children and adolescents remains more mixed.

In view of these gaps, this study conducted a rigorous systematic review and meta-analysis to quantitatively evaluate the overall association between screen time and childhood ADHD risk, and focused on identifying high-risk populations and high-risk exposure patterns through subgroup analyses stratified by age and media type. The study aims to provide evidence-based support for the early prevention of childhood ADHD and the protection of neurodevelopment, and also to offer references for families, educational institutions and public health departments to formulate scientific strategies for children's digital media management.

## 2 Materials and methods

This systematic review and meta-analysis was conducted in strict accordance with the PRISMA 2020 statement (11). The review protocol was not prospectively registered; this limitation is addressed further in the Discussion section.

### 2.1 Search strategy and selection criteria

We systematically searched five major English databases, including PubMed, Embase, The Cochrane Library, Web of Science and PsycINFO, with the search time range from the establishment of each database to January 31, 2025. Search terms combined free-text words and subject headings (including MeSH and Emtree terms where applicable). The core search strategy was as follows: (“Screen time” OR “Electronic media” OR “Television” OR “Smartphone” OR “Tablet” OR “Computer use”) AND (“Attention-Deficit/Hyperactivity Disorder” OR “ADHD” OR “Hyperactivity” OR “Inattention”) AND (“Children” OR “Adolescents” OR “Pediatric” OR “Preschool”). Full database-specific search strings are provided in Supplementary Table 1. No language restrictions were applied at the search stage, though only English-language peer-reviewed publications were ultimately included.

Studies were eligible for inclusion if they met the following criteria: they were observational studies (cross-sectional, case-control, or cohort studies) assessing the association between screen time and ADHD; they enrolled children and adolescents under 18 years of age without underlying neurological comorbidities such as congenital neurodevelopmental defects or brain injury; they clearly recorded and quantified screen time, with distinguishable “excessive/high exposure” and “low/no exposure” groups; they assessed ADHD via standardized clinical diagnostic criteria (DSM-5, ICD-10) or via validated symptom scales (such as the CBCL scale, Conners scale, or SNAP-IV scale); and they reported extractable or convertible effect size data in the form of OR values and 95% CI. Studies focusing exclusively on children with autism spectrum disorder (ASD) or other neurodevelopmental conditions were excluded, as were studies that included mixed neurotypical and neurodivergent samples without reporting stratified effect sizes for neurotypical children.

Exclusion criteria were applied as follows: non-original studies including reviews, case reports, conference abstracts, animal experiments, and *in vitro* studies were excluded; duplicate publications, studies with overlapping data, and studies with incomplete effect size data for which no valid response was received from corresponding authors were excluded; follow-up studies of children already diagnosed with ADHD at baseline were excluded; and studies without clear quantitative standards for screen time or non-standardized ADHD assessment tools were excluded.

### 2.2 Data extraction and quality assessment

Two researchers completed literature screening and data extraction using an independent, duplicate approach, with discrepancies resolved through consultation with a third senior researcher. EndNote X9 (Clarivate Analytics, Philadelphia, PA, USA) was used for reference management and deduplication. The literature screening process proceeded in stages: database retrieval, deduplication, title/abstract screening, full-text screening, and final inclusion. The references of included studies were also hand-searched to identify additional eligible studies. A standardized data extraction form was developed in Microsoft Excel 2021 (Microsoft Corp., Redmond, WA, USA) to extract core information including basic study details, population characteristics, exposure information, outcome measures, adjusted confounding factors, and statistical parameters.

The ROBINS-I (Risk of Bias in Non-randomized Studies - of Interventions) tool was used to evaluate the methodological quality and risk of bias of the included observational studies (12). The evaluation covered seven core bias domains: D1 confounding bias, D2 participant selection bias, D3 intervention classification bias, D4 deviation from intended interventions bias, D5 missing data bias, D6 outcome measurement bias, and D7 selective reporting bias. Each domain was independently rated by two reviewers as low, moderate, serious, critical, or unclear risk, following official ROBINS-I signaling questions and rating criteria. The overall risk of bias for each study was determined by the highest risk rating across any single domain, per standard ROBINS-I guidance. Disagreements were resolved through discussion with a third senior reviewer.

### 2.3 Statistical analysis

All statistical analyses were performed using Stata 17.0 software (StataCorp LLC, College Station, TX, USA), with a test level of *α*=0.05.

For studies reporting outcome indicators as continuous variables, the Chinn conversion rule was applied to convert standardized mean differences (SMD) into log odds ratios (lnOR) to unify effect sizes across all studies (13); This approach allows pooling of both dichotomous diagnostic outcomes and continuous symptom scores on a common scale, which is standard practice in meta-analyses combining multiple outcome formats. Final results are reported as OR values with 95% CI.

Cochrane's Q test and I^2^ statistic were used to assess inter-study heterogeneity. I^2^ < 25% was considered low heterogeneity, 25%≤I^2^ ≤ 50% moderate heterogeneity, and I^2^ > 50% high heterogeneity. A *P*-value threshold of <0.10 was used for the Q test to determine significant heterogeneity, consistent with standard meta-analytic practice; this more lenient threshold accounts for the low statistical power of the Q test when the number of included studies is small (14); Given expected inter-study heterogeneity, the DerSimonian-Laird random-effects model was used for all primary analyses to calculate pooled OR values and 95% CI (15). For subgroups with I^2^ < 25%, a fixed-effects model was also run as a supplementary verification analysis.

Stratified subgroup analyses were conducted to explore sources of heterogeneity, with formal between-subgroup heterogeneity tests performed to assess statistically significant differences across groups. Subgroups were defined by age and media type, with classification rationales as follows:

Age subgroups: Children aged 0–6 years were classified as preschoolers, and children over 6 years as school-age and adolescents. This division aligns with standard developmental staging, corresponds to the typical age of primary school entry globally, and matches age stratification used in WHO screen time guidelines and prior research.

Media type subgroups: Three categories were defined:

Passive TV/video viewing: Linear, pre-recorded screen content requiring no active user input, including broadcast television, streaming programs, and pre-recorded animations.
Interactive digital media: Screen-based activities requiring real-time user input and active cognitive engagement, including educational apps, video games, and interactive learning software (encompassing both educational and entertainment content).
General/mixed screen time: Total screen time across all device types and content formats, for which passive and interactive media were not distinguished separately.

Sensitivity analysis was conducted using the leave-one-out method, in which each included study is excluded in turn and the remaining studies are re-pooled to evaluate the influence of individual studies on the overall effect and the robustness of core conclusions. Publication bias was assessed visually via Begg's funnel plot and quantitatively via Egger's linear regression test and Kendall's *τ* test, with *P* > 0.05 used as the threshold for no statistically significant publication bias (16).

One study (Lingineni 2012) (17) reported separate effect sizes for passive TV and interactive computer use, which were included in the respective media type subgroups without double-counting the overall sample.

## 3 Results

### 3.1 Literature screening results and basic characteristics of included studies

A total of 2,376 relevant studies were retrieved from the databases. After de-duplication with EndNote X9, 1,892 studies remained. 1,835 studies were excluded after title/abstract initial screening, and 57 studies were subject to full-text re-screening. Finally, 47 studies were excluded (exclusion reasons: 21 with incomplete data, 12 with inconsistent study types, 8 with ambiguous definition of exposure/outcome, 6 with duplicate publications), and 10 eligible observational studies were finally included. The study selection process is presented in Figure 1, following PRISMA 2020 guidelines.

**Figure 1:**
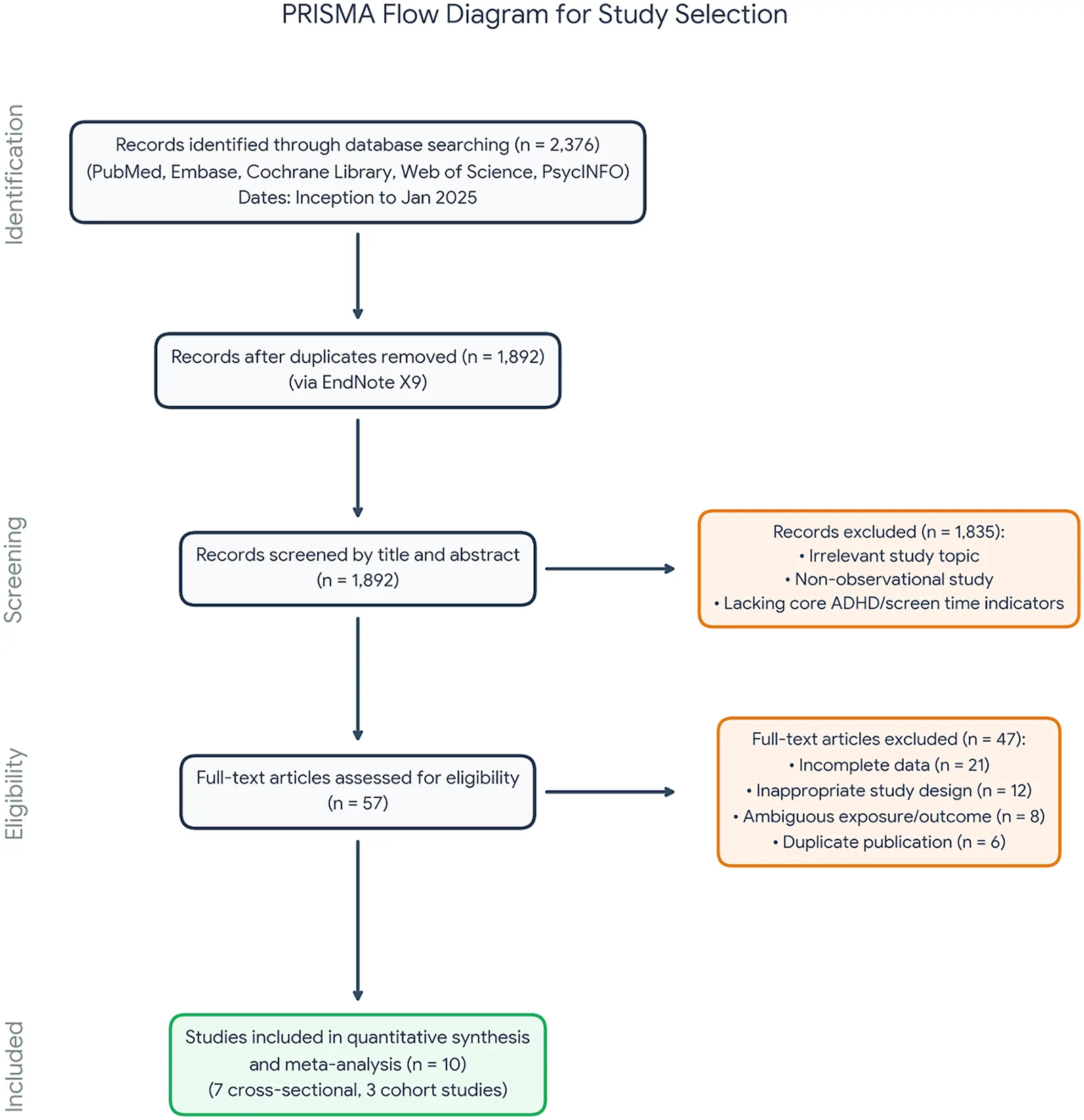
PRISMA 2020 flow diagram of the study selection process.

All the 10 included studies were English publications from 2012 to 2025, covering North America, Europe, Asia and Oceania; among them, there were 7 cross-sectional studies and 3 cohort studies, with a total sample size of 232,125 cases, and the age range of the study population was 0–17 years old. The exposure types included general/mixed screen time (7 studies), passive TV/video viewing (2 studies) and interactive digital media (2 studies). Of note, the study by Lingineni et al. (17) reported separate effect sizes for passive TV viewing and interactive computer use, and was thus included in both the passive and interactive media subgroups without double-counting the overall sample. The main ADHD assessment tools included the Strengths and Difficulties Questionnaire (SDQ, 3 studies), Child Behavior Checklist (CBCL, 2 studies), parent-reported clinical diagnosis (2 studies), Conners scale (1 study), ADHD Rating Scale-IV (1 study), and self-rated symptoms (1 study); all studies adjusted at least one confounding factor such as gender, age and family socioeconomic status. Basic characteristics of included studies are summarized in Table 1.

**Table 1:** Basic characteristics of the included studies.

First Author (Year)	Study Design	Sample Size	Age	Assessment Method	Screen Time Criteria (High Exposure)	ADHD Assessment Criteria	Screen Media Type	ROBINS-I Overall Risk of Bias
Nagata (2024) (18)	Prospective cohort	9,538	9–10 yrs	Self-reported & Parent survey	Continuous (per 1-h increase)	CBCL (Parent-reported)	General/Mixed	Low
Qu (2023) (19)	Cross-sectional	101,350	0–17 yrs	Parent survey (NSCH)	≥ 4 h/day	Parent-reported clinical diagnosis	General/Mixed	Moderate
Liu (2021) (20)	Birth cohort	2,492	4 yrs	Parent questionnaire	> 2 h/day	SDQ (Abnormal score)	General/Mixed	Moderate
Ra (2018) (7)	Longitudinal cohort	3,051	15–16 yrs	Self-reported questionnaire	High-frequency digital media use	Self-rated ADHD symptoms	Interactive/Digital	Moderate
Lingineni (2012) (17)	Cross-sectional	68,634	5–17 yrs	Parent survey (NSCH)	≥ 1 h/day	Parent-reported clinical diagnosis	Passive TV/Interactive PC	Moderate
Wu (2025) (21)	Cross-sectional	41,494	4–6 yrs	Parent questionnaire	> 120 min/day	SDQ (Total score ≥ 6)	Passive video	Moderate
Zhou (2023) (22)	Cross-sectional	2,452	Preschool	Parent questionnaire	> 1 h/day	Conners Child Behavior Scale	General/Mixed	Moderate
Xie (2020) (23)	Cross-sectional	1,897	3–6 yrs	Parent questionnaire	> 60 min/day	CBCL (Externalizing behavior)	General/Mixed	Moderate
Moulin (2022) (24)	Cross-sectional	432	Mean 6.8 yrs	Online parent questionnaire	> 1 h/day	SDQ (Abnormal score)	General/Mixed	Serious
Tong (2016) (25)	Cross-sectional	785	9–13 yrs	Parent questionnaire	Continuous variable	ADHD Rating Scale-IV (Home)	General/Mixed	Serious

ROBINS-I overall risk of bias, low, moderate, serious. Two studies (Moulin 2022, Tong 2016) were rated as serious risk mainly due to participant selection bias and outcome measurement bias.

### 3.2 Risk of bias assessment results of included studies

The distribution of risk of bias across the seven ROBINS-I domains is shown in Figure 2, with proportions calculated based on individual study ratings for each domain. Individual study-level risk of bias ratings are presented in Figure 3. In the D4 (deviation from intended interventions) domain, all studies were rated as low risk (100%). D1 (confounding bias), D5 (missing data bias), and D7 (selective reporting bias) were predominantly moderate risk (70%, 80%, and 70% respectively). D2 (participant selection bias) and D6 (outcome measurement bias) had relatively higher proportions of serious risk (40% and 30% respectively). D3 (intervention classification bias) was rated moderate risk for all studies (100%).

**Figure 2:**
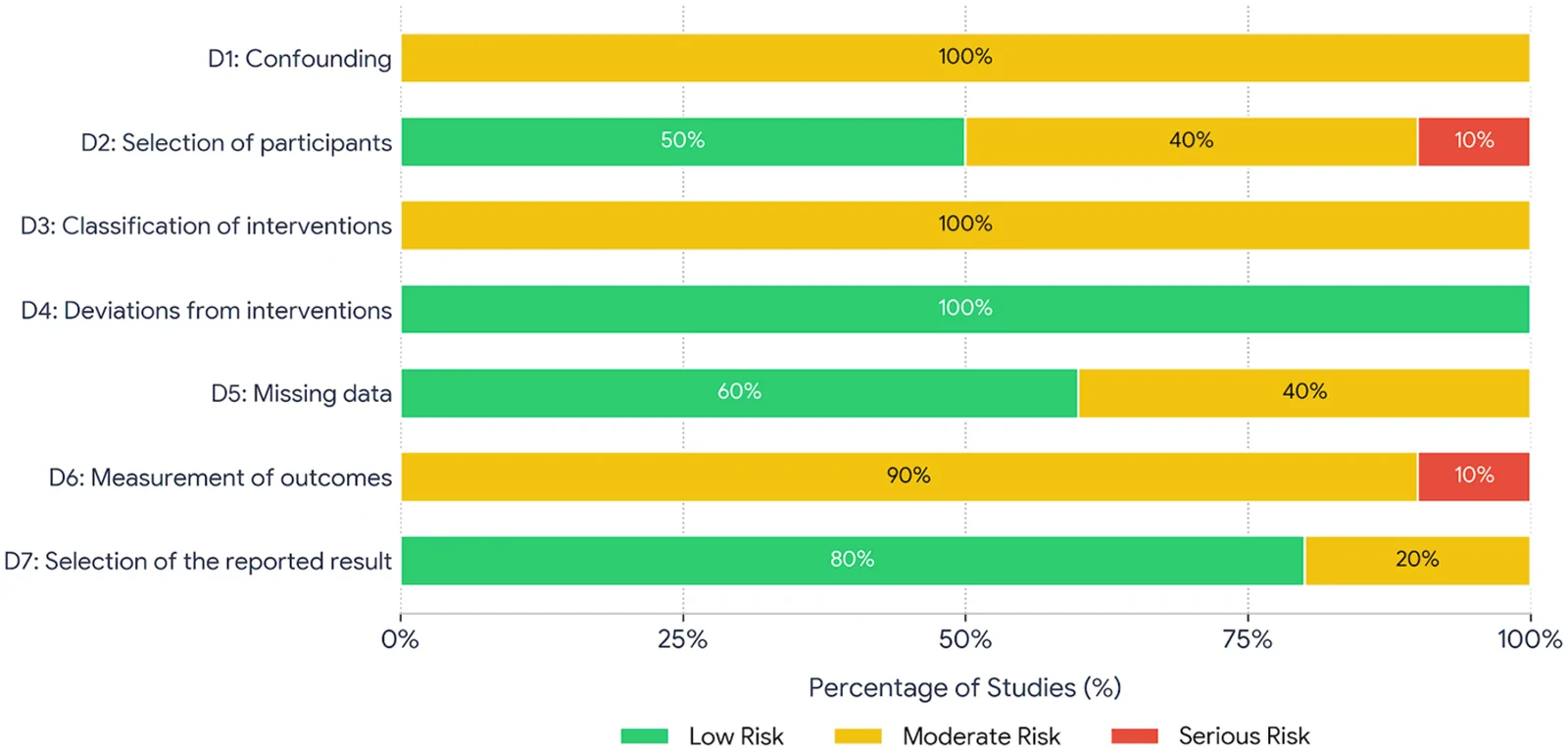
Summary of ROBINS-I risk of bias across all included studies (percentage distribution).

**Figure 3:**
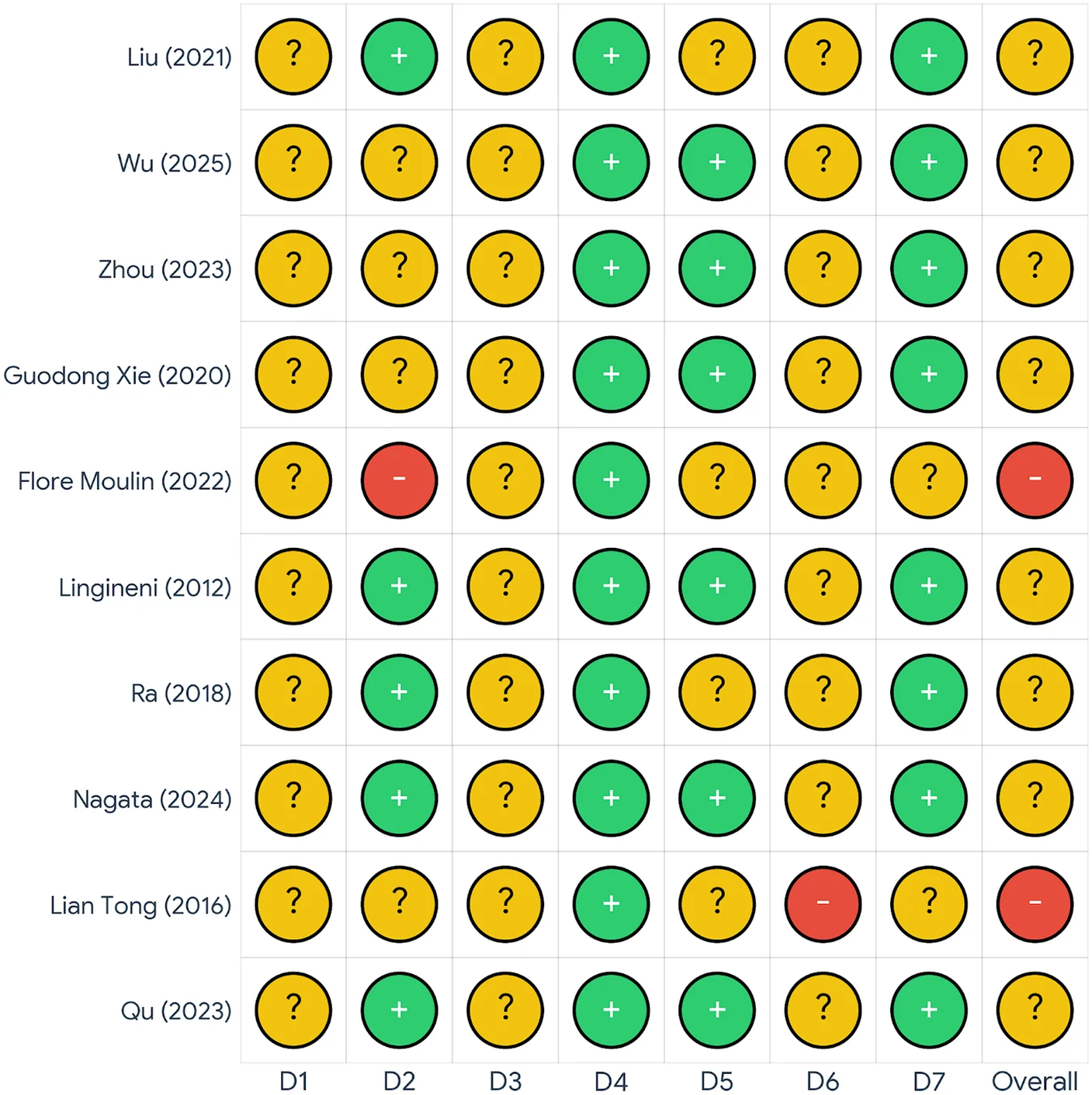
ROBINS-I risk of bias traffic light plot for individual included studies.

Overall, 1 study (18) was rated low risk of bias, 7 studies (7, 17, 19–23) were moderate risk, and 2 studies (24, 25) were rated serious risk, primarily due to participant selection bias and outcome measurement bias. On the whole, the included studies had an overall low-to-moderate risk of bias, and their methodological quality was within an acceptable range.

### 3.3 Main effect analysis

Pooled results from the random-effects model showed that excessive screen time was significantly positively associated with the risk of ADHD in children, with a pooled OR of 1.56 (95% CI: 1.31–1.87, *P* < 0.001). Heterogeneity test showed that there was high statistical heterogeneity among studies (Q = 344.51, *P* < 0.001, I^2^ = 97.4%), The forest plot for the main effect is shown in Figure 4.

**Figure 4:**
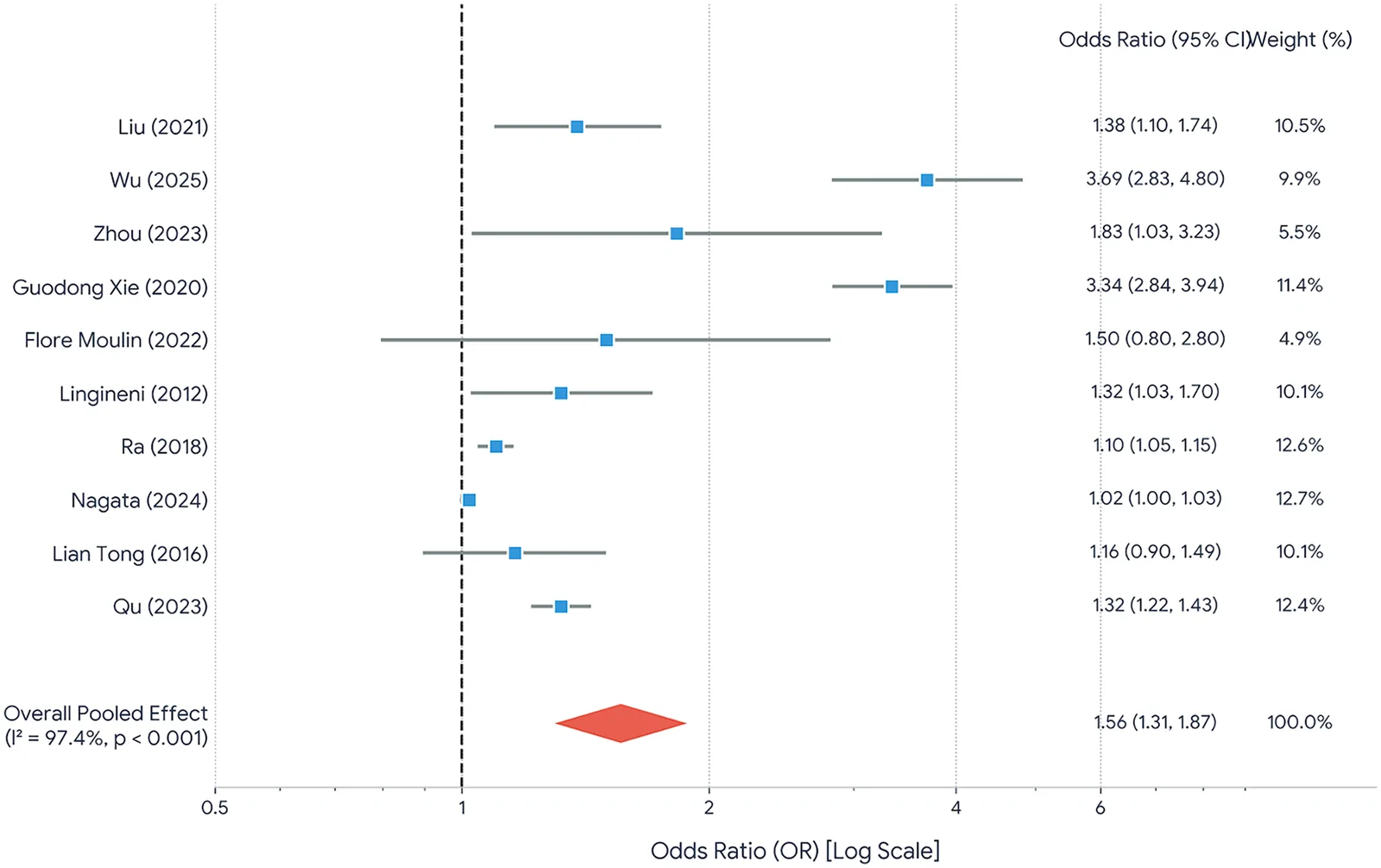
Forest plot of the main effect meta-analysis for the association between excessive screen time and childhood ADHD risk.

### 3.4 Subgroup analysis results

#### 3.4.1 Age-based subgroup analysis

Age-stratified subgroup analysis revealed a statistically significant difference in the association between screen time and ADHD risk across age groups. Age-stratified subgroup analysis revealed a statistically significant difference in the association between screen time and ADHD risk across age groups, as confirmed by the formal between-subgroup heterogeneity test (*Q*_between_ = 7.56, *P* = 0.006). Both subgroups exhibited high within-group heterogeneity (preschool subgroup: *Q* = 187.23, *I*^2^ = 98.4%, *P* < 0.001; school-age/adolescent subgroup: *Q* = 149.67, *I*^2^ = 96.7%, *P* < 0.001):

1. Preschool children (0–6 years old): 4 studies were included, with a pooled OR = 2.40, 95% CI: 1.44–3.98, *P* < 0.001, indicating a markedly stronger magnitude of association in this age group;
2. School-age children and adolescents (>6 years old): 6 studies were included, with a pooled OR = 1.16, 95% CI: 1.05–1.29, *P* = 0.004, indicating a statistically significant but substantially smaller magnitude of association relative to preschool children. The forest plot for age subgroup analysis is shown in Figure 5.

**Figure 5:**
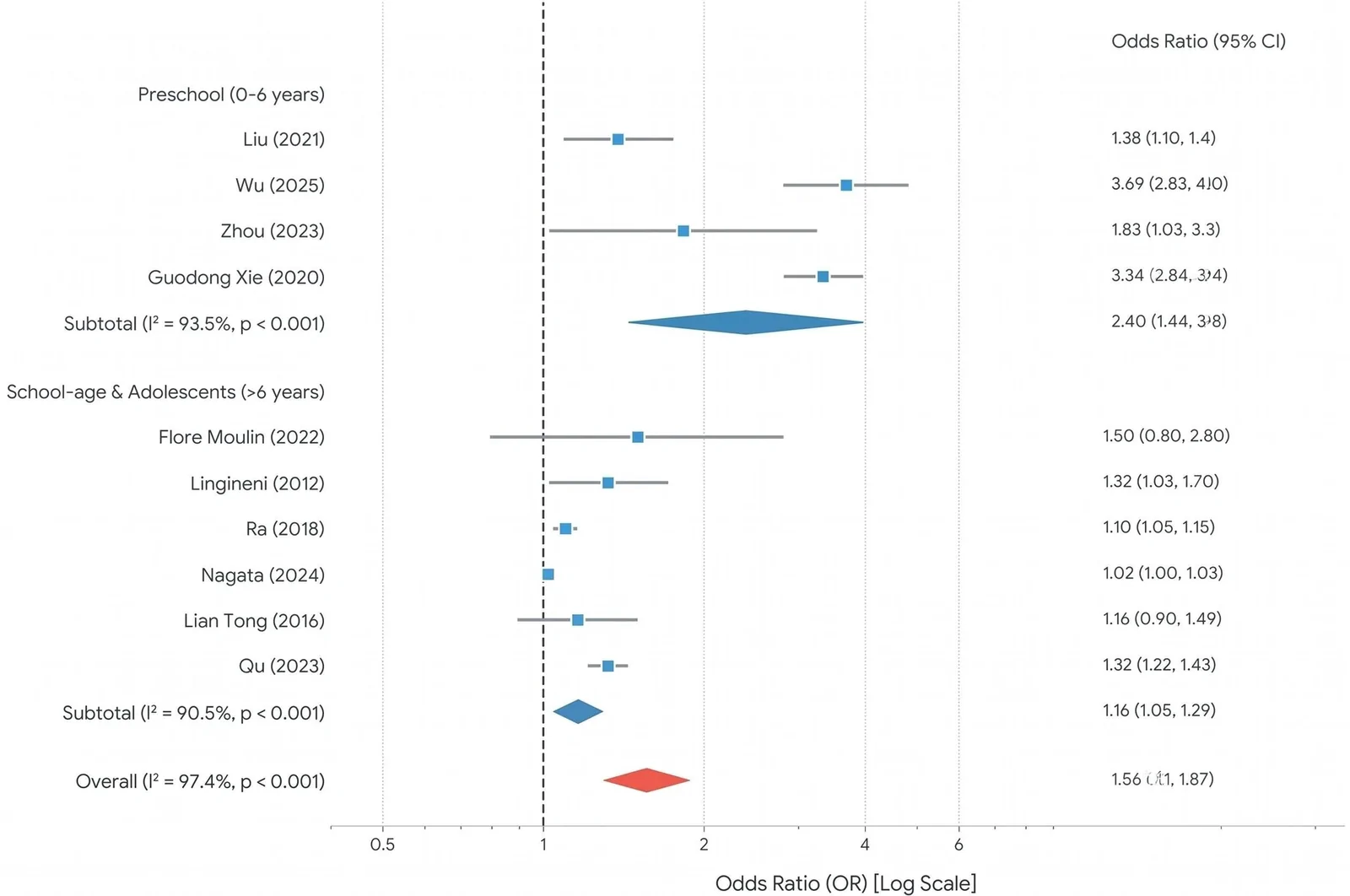
Forest plot of the age-based subgroup analysis.

#### 3.4.2 Media type-based subgroup analysis

Media type-stratified subgroup analysis showed a trend toward differing magnitudes of association with ADHD risk across screen media types, although the between-group difference did not reach statistical significance (*P* > 0.05). Note: For this media type-specific analysis, the study by Lingineni (17) was split into two independent data points (passive TV and interactive computer) to capture distinct media effects. This resulted in 11 data points for the subgroup analysis, with an adjusted overall pooled OR of 1.50 (95% CI: 1.27∼1.78, I^2^ = 97.1%). This value differs slightly from the primary overall OR (1.56) which was based on 10 independent studies without data splitting.

1. Passive TV/video viewing: 2 studies were included, with a pooled OR = 2.20, 95% CI: 0.81–6.03, and substantial within-group heterogeneity (*Q* = 12.45, *I*^2^ = 91.9%, *P* = 0.002). The point estimate of the OR was numerically larger than that for other media types, but the wide confidence interval spanning the null value reflected limited statistical power in this subgroup, driven by the small number of included studies and high within-group heterogeneity;
2. Interactive digital media: 2 studies were included, with a pooled OR = 1.10, 95% CI: 1.05–1.15, *P* < 0.001, and no inter-study heterogeneity (*I*^2^ = 0%, *Q* = 0.32, *P* = 0.572). The magnitude of the association was relatively small;
3. General/mixed screen time: 7 studies were included, with a pooled OR = 1.52, 95% CI: 1.12–2.05, *P* < 0.01, and high within-group heterogeneity (*Q* = 128.91, *I*^2^ = 95.3%, *P* < 0.001). The effect size was between the passive and interactive media subgroups.

The forest plot for media type subgroup analysis is shown in Figure 6.

**Figure 6:**
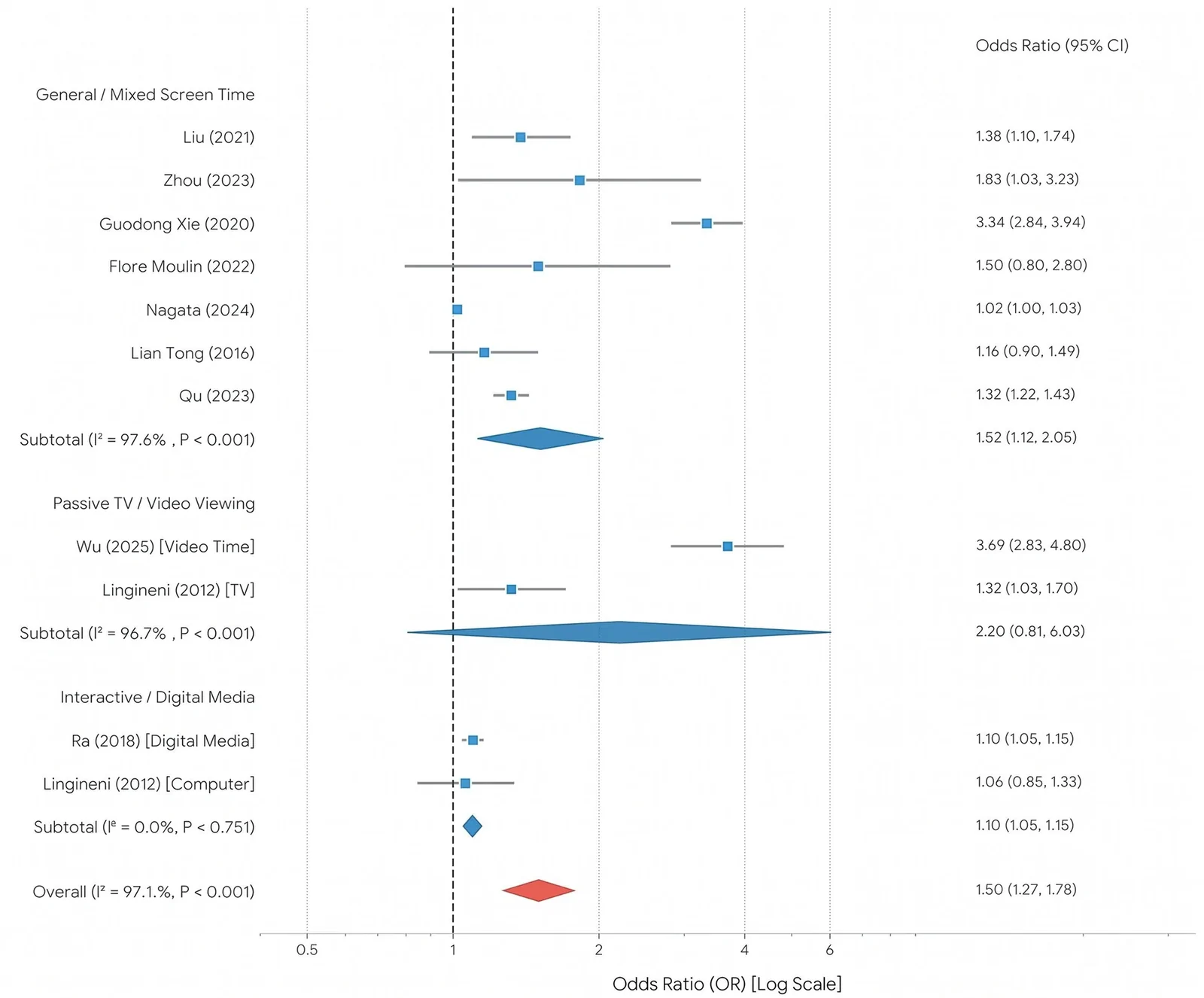
Forest plot of the media type-based subgroup analysis.

### 3.5 Sensitivity analysis and publication bias assessment

#### 3.5.1 Sensitivity analysis

Leave-one-out sensitivity analysis showed that after sequentially excluding each individual study, pooled OR values ranged from 1.38 to 1.67, and all 95% CIs excluded the null value of 1, with all *P* values <0.05. After excluding the 2 studies rated as having serious risk of bias (24, 25), the pooled OR remained 1.52 (95% CI: 1.28–1.80). When excluding all cross-sectional studies and pooling only the 3 prospective cohort studies (7, 18, 20), the pooled OR was 1.48 (95% CI: 1.21–1.82). These results indicate that the core findings are robust and not driven by any single study or study design. The leave-one-out sensitivity analysis forest plot is shown in Figure 7.

**Figure 7:**
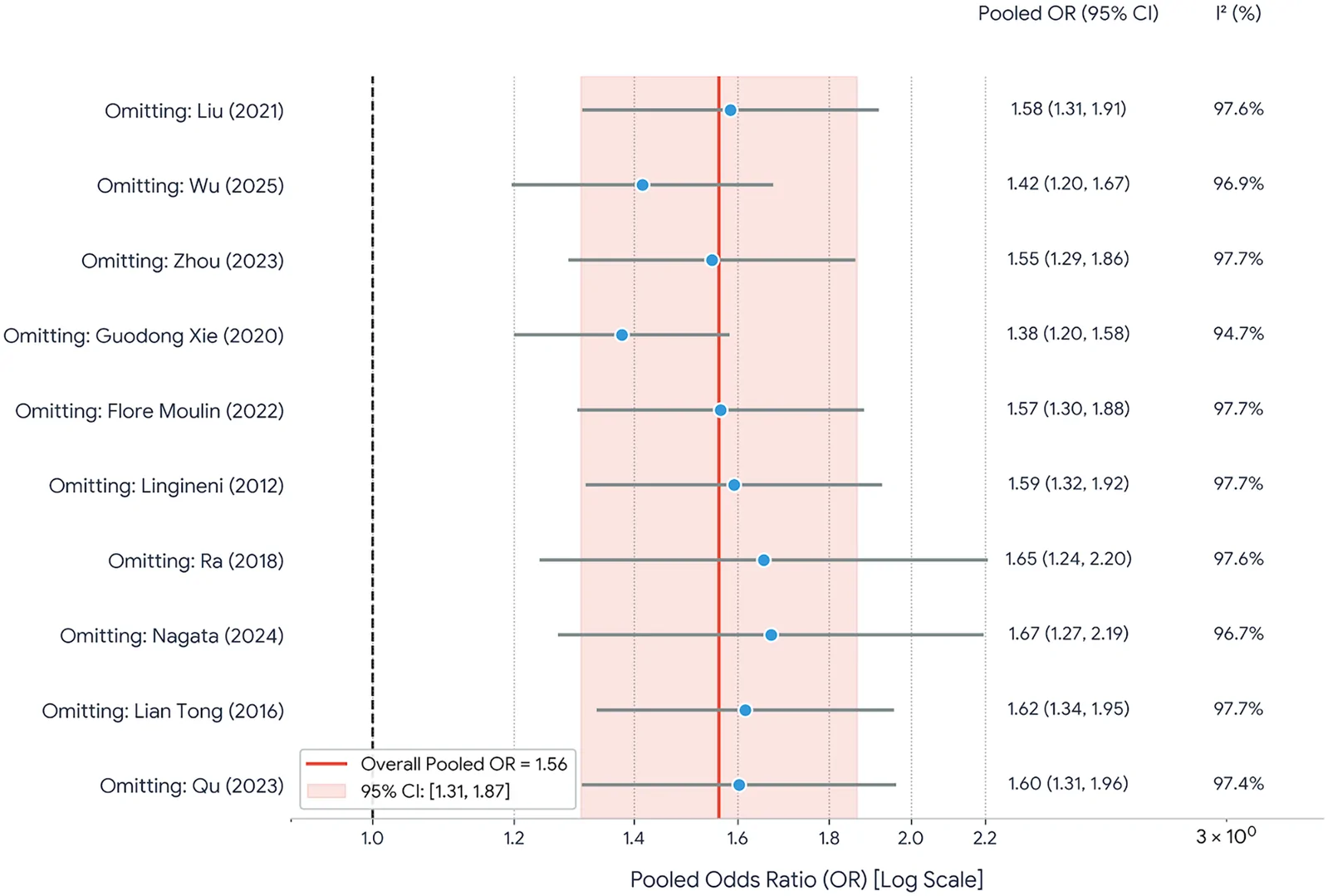
Forest plot of the leave-one-out sensitivity analysis.

#### 3.5.2 Publication bias assessment

Publication bias was assessed visually via Begg's funnel plot (Figure 8) and quantitatively via Begg's test and Egger's linear regression test. The funnel plot showed roughly symmetric distribution of study points. Quantitative tests yielded inconsistent results: Kendall's *τ* = −0.067, *P* = 0.862 for Begg's test, which did not reach statistical significance; however, the more sensitive Egger's test detected potential small-study effects (intercept = 5.037, *P* = 0.020). These findings suggest the possibility of publication bias or small-study effects, which should be considered when interpreting the pooled effect size.

**Figure 8:**
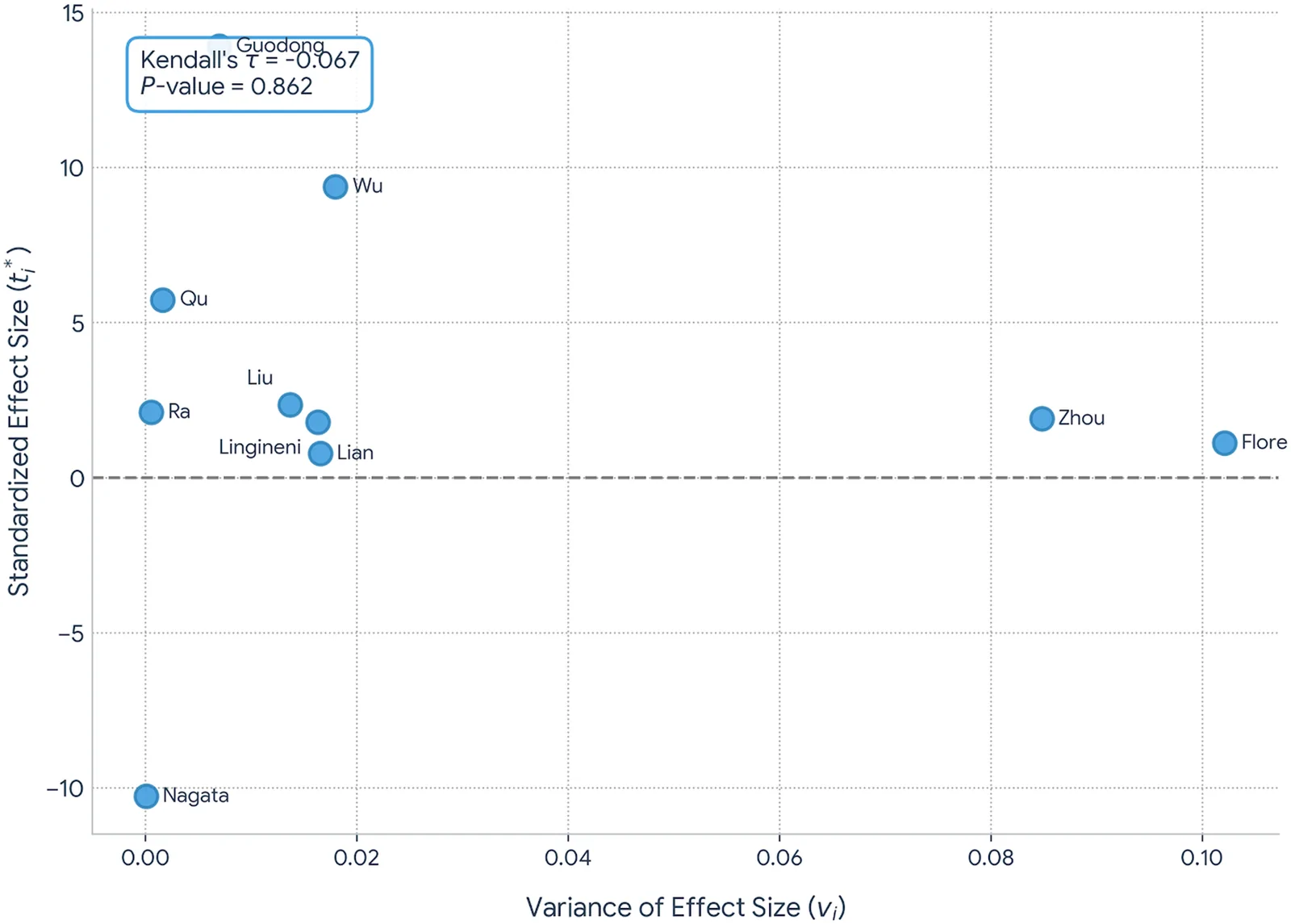
Begg's funnel plot for the assessment of publication bias.

## 4 Discussion

Based on the systematic synthesis of 10 observational studies with overall low-to-moderate risk of bias, this meta-analysis found a significant positive association between excessive screen time and the risk of ADHD in children (pooled OR = 1.56). The study systematically revealed that this association exhibits distinct age-specific and media type-specific patterns: preschool children (0–6 years old) show a stronger association with ADHD risk, and passive TV/video viewing has a higher point estimate of risk than interactive digital media. These findings extend existing evidence on screen exposure and child neurodevelopment, and provide key evidence-based support for targeted digital media management and early ADHD prevention. It should be noted that all findings are based on observational evidence and cannot establish definitive causal relationships.

### 4.1 Age specificity: neurodevelopmental vulnerability of preschool children

This study confirmed via formal between-subgroup heterogeneity testing that the effect size of the association between screen exposure and ADHD risk in preschool children (OR = 2.40) was significantly higher than that in school-age children and adolescents (Q_between_ = 7.56, *P* = 0.006), which is highly consistent with the plasticity law of brain development. The age of 0–6 years is a critical window for children's brain neurodevelopment. During this period, the prefrontal cortex (PFC)—the core brain area responsible for attention allocation, impulse control, executive function and working memory—is in the peak period of synaptogenesis and neural circuit construction (26), with extremely strong neural plasticity, but at the same time it is highly sensitive to external environmental stimuli.

Several theoretical mechanisms have been proposed to explain the greater vulnerability of preschool-aged children, though these remain to be directly validated in human studies. First, the prefrontal neural circuits of preschool children are not yet fully developed, and they lack mature ability to filter and inhibit irrelevant sensory information. It is hypothesized that fast-paced, high-stimulation screen content may produce a “sensory overload” effect: long-term exposure to such stimuli may lead the brain to adapt by down-regulating dopamine receptor sensitivity (27). Eventually, children's ability to sustain attention on low-stimulation, real-world tasks that require active attention—such as reading, classroom learning, and parent-child communication—may decline, producing ADHD-like symptoms such as inattention and hyperactivity. Second, screen exposure in preschool children may exert a significant “displacement effect" (28), taking time that would otherwise be spent on outdoor activities, physical play, and face-to-face social interaction. These activities are necessary for prefrontal cortex development and executive function building, and their displacement may negatively affect the development of children's attention regulation and behavioral inhibition abilities.

In contrast, the brain neural circuits of school-age children and adolescents have basically matured, the function of the prefrontal cortex tends to be stable, and their tolerance and regulation ability to external stimuli are significantly improved. Accordingly, the magnitude of the association between screen exposure and ADHD risk is smaller in this age group (OR = 1.16). Nevertheless, this result still indicates a need for reasonable management of screen time in school-age children and adolescents, as even mature brains may experience elevated ADHD risk with long-term excessive screen exposure.

It is important to note that the mechanisms described above represent theoretical explanatory frameworks and were not directly evaluated in the included studies. Recent narrative reviews have also highlighted potential structural and functional brain changes associated with early screen exposure, including reduced prefrontal gray matter volume and altered functional connectivity in attention networks, which may underlie the observed associations (29). Further neuroimaging research is needed to validate these pathways. Additionally, bidirectional relationships between screen use and ADHD symptoms have been proposed, encompassing both a media effect pathway and a selection effect pathway, and both may operate simultaneously across development (29).

### 4.2 Media type differences: active vs. passive attention

Another notable observation is the trend toward a stronger association between passive TV/video viewing and ADHD risk compared with interactive digital media. The point estimate for passive TV/video viewing (OR = 2.20) was numerically higher than that for interactive digital media (OR = 1.10), but the between-group difference did not reach statistical significance.

The non-significant between-group difference is likely attributable to low statistical power in the passive video subgroup, which included only 2 studies and had a very wide confidence interval. In addition, inconsistent classification criteria for media content across studies may have introduced misclassification bias and further attenuated the true between-group difference. This finding therefore remains preliminary and requires confirmation in larger, specifically designed studies with standardized content classification.

This result can be reasonably explained by the “pacing hypothesis” and differences in cognitive engagement (30). Passive TV/video content—especially children's animations and entertainment programs—typically uses fast cuts, rapid narrative shifts, and high color contrast to maintain attention through external, forced stimulation. This form of attention is classified as “passive attention”, which does not require children to actively mobilize cognitive resources for attention allocation and maintenance (10). Habituation to this low-effort passive attention mode may significantly inhibit the development of children's active attention, making it difficult to sustain concentration on low-stimulation tasks that require independent attention regulation.

In contrast, interactive digital media such as educational games and learning applications require active cognitive engagement from users, including logical judgment, operational feedback, and problem solving. This process engages children's active attention and executive functions, which may partially attenuate the observed association between screen exposure and ADHD risk (31). Accordingly, its association with ADHD risk is substantially weaker.

This finding challenges the monolithic view that “all screen time is equally harmful”, and suggests that the quality of children's screen use may be more important than the quantity. Compared with simply limiting total screen time, guiding children toward screen activities with high cognitive engagement and reducing unstructured passive TV/video viewing may be a more promising strategy for supporting healthy neurodevelopment.

### 4.3 Addressing controversies and heterogeneity

Core controversies regarding the association between screen time and ADHD center on causal direction and confounding (8). The 3 cohort studies included in this review all used prospective designs, and all found that high baseline screen time predicted subsequent ADHD symptoms, providing longitudinal evidence consistent with a temporal sequence from screen exposure to later symptoms (7, 18, 20). Additionally, all included studies adjusted for key confounding factors such as gender, age, family socioeconomic status, and parenting style, and our meta-analysis further accounted for inter-study heterogeneity. Taken together, these findings support the existence of an independent positive association between screen time and ADHD risk.

Regarding the “reverse causality” hypothesis—that children with hyperactive tendencies are more likely to be drawn to screens—our sensitivity analysis showed that even when excluding cross-sectional studies (which are most vulnerable to reverse causality) and pooling only cohort study results, a significant association remained (OR = 1.48, 95% CI: 1.21–1.82). This suggests that reverse causality is not the sole explanation for the observed association. However, a growing body of evidence supports bidirectional relationships, encompassing both a media effect pathway (screen exposure contributing to attentional difficulties) and a selection effect pathway (children with attentional difficulties seeking out more high-stimulation screen content) (29). Both directions likely operate simultaneously, and further long-term longitudinal research is needed to disentangle these pathways. The present meta-analysis found very high between-study heterogeneity (*I*^2^ = 97.4%), which has critical implications for the interpretation of pooled estimates. Several factors likely contribute to this substantial variability. First, differences in study design (cross-sectional vs. prospective cohort) may produce different effect size estimates, with cross-sectional studies generally reporting larger associations than prospective cohorts. Second, outcome measures vary across studies, ranging from formal clinical diagnosis to parent- or self-rated symptom scales; these different outcomes capture different severity levels of ADHD-related impairment and are not directly interchangeable, which is a major source of statistical heterogeneity. Third, definitions of “excessive screen time” vary widely across studies, with thresholds ranging from 1 to 4 h per day, and exposure assessment methods also differ (e.g., daily average vs. weekday only). Fourth, the composition of media types differs across general/mixed screen time studies, mixing passive and interactive content to varying degrees. Finally, geographic and cultural differences may modify the association between screen time and developmental outcomes.

Given the extremely high heterogeneity, the pooled OR value should be interpreted primarily as an indicator of the direction of the association, rather than a precise estimate of effect magnitude. Although sensitivity analyses confirmed that the direction and statistical significance of the association remain robust, the absolute effect size may vary substantially across specific populations and study contexts.

In terms of bias control, this study used independent, duplicate screening and data extraction to reduce human bias. The ROBINS-I tool was used for comprehensive risk of bias assessment, and included studies were of overall acceptable methodological quality. While Egger's test indicated potential small-study effects, sensitivity analyses confirmed that core conclusions remain robust even after excluding potential outliers.

### 4.4 Strengths and limitations

#### 4.4.1 Strengths

This study has several notable strengths. First, it strictly followed the PRISMA 2020 statement (11), with a comprehensive search strategy, rigorous screening and data extraction procedures, and robust bias control measures, yielding relatively reliable evidence. Second, it is the first meta-analysis to systematically explore the moderating roles of both age and media type in the association between screen time and ADHD, identifying high-risk populations and exposure patterns and addressing key gaps in prior reviews. Third, it used the Chinn conversion rule to unify effect sizes across continuous and categorical outcomes (13), applied the DerSimonian-Laird random-effects model for pooling (15), and conducted multi-dimensional sensitivity analyses, with rigorous statistical methods and robust conclusions. Fourth, the included studies encompass a large total sample size and cover multiple global regions, supporting good external validity.

#### 4.4.2 Limitations

Several limitations should be acknowledged. First, most included studies used cross-sectional designs (7/10), with only 3 prospective cohort studies, which precludes definitive confirmation of a causal relationship between screen time and childhood ADHD risk. Second, the definition of “excessive screen time” was inconsistent across studies, with thresholds ranging from 1 to 4 h per day, and specific contexts and frequencies of use were not further stratified, contributing to residual statistical heterogeneity. Third, the media type subgroup analysis had notable methodological limitations. The small number of eligible studies (only 2 each for passive TV/video and interactive digital media) resulted in insufficient statistical power, which may have prevented detection of a true between-group difference. In addition, heterogeneous definitions and classification methods for screen content types across included studies may have introduced classification bias and affected the reliability of subgroup comparisons. Findings related to media type differences therefore require validation in larger, more geographically diverse cohorts with standardized content classification frameworks. Fourth, the review only included English-language peer-reviewed publications, which may introduce language bias. Fifth, dose-response meta-analysis was not feasible due to variable exposure categorization across studies and a lack of multi-level dose data. Sixth, the review protocol was not prospectively registered in a platform such as PROSPERO, which is a methodological limitation. Finally, the included studies used a mix of clinical diagnoses and symptom scale outcomes, which may not be directly comparable and may contribute to observed heterogeneity.

### 4.5 Implications for public health and clinical practice

The findings of this meta-analysis provide preliminary evidence-based support for early ADHD prevention, neurodevelopmental protection, and scientific digital media management, with implications for pediatric clinical practice, public health policy, family parenting, and educational interventions. All recommendations below should be interpreted as provisional guidance based on observational evidence, not definitive policy.

In pediatric clinical settings, screen use patterns should be integrated into routine neurodevelopmental assessments, with targeted attention to preschool children aged 0–6 years. Children with high screen exposure should be monitored and provided with early follow-up, and parents should receive personalized guidance on age-appropriate screen use practices.

For public health policy, guidelines for children's digital media use should be refined, with a focus on reducing passive screen exposure in preschoolers aged 0–6 years. Existing recommendations for zero screen time for children under 2 years and limited daily screen time for those aged 3–6 years should be reinforced. Additionally, tiered management of children's digital content should be promoted, encouraging the development of high-cognitive-engagement interactive educational materials and discouraging fast-paced, low-nutrition passive children's video content.

In family parenting, parents should shift from a sole focus on limiting screen time to dual management of both quantity and quality. Unsupervised passive TV/video viewing should be reduced, and parent-child interactive screen use such as educational games and learning apps should be encouraged. Excessive screen time should be replaced with outdoor activities, parent-child reading, and physical play to create a low-screen family environment.

Finally, kindergartens and primary schools should strengthen management of children's screen use, minimize passive screen exposure in teaching processes, increase offline interactive teaching and outdoor activities, and implement attention training programs to enhance children's active attention and executive functions.

## 5 Conclusions

In conclusion, excessive screen exposure is significantly associated with elevated risk of attention-deficit/hyperactivity disorder (ADHD) in children, and this association exhibits distinct age-specific and media type-specific patterns. Preschool children aged 0–6 years appear to be particularly vulnerable to screen-related effects, with a statistically significant difference in effect magnitude relative to older children. Passive TV/video viewing shows a trend toward a higher point estimate of risk than interactive digital media requiring active cognitive engagement, though the between-group difference did not reach statistical significance and requires further confirmation in larger studies.

The preschool period is both a critical window for nervous system development and an optimal timing for screen exposure intervention. Public health guidelines, pediatric clinical practice, and family parenting should collaborate to prioritize reduction of early passive screen exposure in children aged 0–6 years, guide children toward high-cognitive-engagement screen use, and replace unstructured screen time with outdoor activities, parent-child interaction, and physical play.

Future research with large, long-term prospective cohorts is needed to clarify causal pathways and establish evidence-based safe screen time thresholds for different age groups and media types.

## Data Availability

The original contributions presented in the study are included in the article/Supplementary Material, further inquiries can be directed to the corresponding author.
