# Supplementary material for: Screen time and attention-deficit/hyperactivity disorder in children: a systematic review and meta-analysis
Source: Front Pediatr. 2026 Sep 28;14:1860549. doi: 10.3389/fped.2026.1860549 (PMC13655039; doi:10.3389/fped.2026.1860549)
Supplement: Supplementary file 1 [file Table1.docx]

Supplementary Table 1

Full search strategies for five electronic databases

Note. All searches were run on January 31, 2025, covering the period from database inception to the search date. No language restrictions were applied at the search stage.

Abbreviations: MeSH = Medical Subject Headings; Emtree = Embase subject headings; ti = title; ab = abstract; TS = topic; DE = descriptor term.

| **Database** | **Full Search Strategy** |
| --- | --- |
| **PubMed (MEDLINE via PubMed)** | #1 "Screen Time"[MeSH Terms] OR "screen time"[tiab] OR "electronic media"[tiab] OR "digital screen"[tiab] OR "sedentary screen use"[tiab] OR "Television"[MeSH Terms] OR television[tiab] OR "smartphone"[tiab] OR "Tablet Computers"[MeSH Terms] OR tablet[tiab] OR "computer use"[tiab] OR "digital media"[tiab]  #2 "Attention Deficit Disorder with Hyperactivity"[MeSH Terms] OR "attention-deficit/hyperactivity disorder"[tiab] OR ADHD[tiab] OR hyperactivity[tiab] OR inattention[tiab] OR "attention problems"[tiab] OR "hyperkinetic disorder"[tiab] OR "executive dysfunction"[tiab]  #3 "Child"[MeSH Terms] OR "Adolescent"[MeSH Terms] OR child*[tiab] OR adolescen*[tiab] OR pediatric[tiab] OR paediatric[tiab] OR preschool*[tiab] OR "young children"[tiab]  #4 #1 AND #2 AND #3 |
| **Embase (via** [**Embase.com**](https://link.wtturl.cn/?target=https%3A%2F%2FEmbase.com&scene=im&aid=582478&lang=zh)**)** | #1 'screen time'/exp OR 'screen time':ti,ab OR 'electronic media':ti,ab OR 'digital screen':ti,ab OR 'sedentary screen use':ti,ab OR 'television'/exp OR television:ti,ab OR 'smartphone':ti,ab OR 'tablet computer'/exp OR tablet:ti,ab OR 'computer use':ti,ab OR 'digital media':ti,ab  #2 'attention deficit hyperactivity disorder'/exp OR 'attention-deficit/hyperactivity disorder':ti,ab OR ADHD:ti,ab OR hyperactivity:ti,ab OR inattention:ti,ab OR 'attention problems':ti,ab OR 'hyperkinetic disorder'/exp OR 'executive dysfunction':ti,ab  #3 'child'/exp OR 'adolescent'/exp OR child*:ti,ab OR adolescen*:ti,ab OR pediatric:ti,ab OR paediatric:ti,ab OR preschool*:ti,ab OR 'young children':ti,ab  #4 #1 AND #2 AND #3 |
| **Cochrane Library (CENTRAL)** | #1 MeSH descriptor: [Screen Time] explode all trees OR screen time:ti,ab OR electronic media:ti,ab OR digital screen:ti,ab OR sedentary screen use:ti,ab OR MeSH descriptor: [Television] explode all trees OR television:ti,ab OR smartphone:ti,ab OR tablet:ti,ab OR computer use:ti,ab OR digital media:ti,ab  #2 MeSH descriptor: [Attention Deficit Disorder with Hyperactivity] explode all trees OR attention-deficit/hyperactivity disorder:ti,ab OR ADHD:ti,ab OR hyperactivity:ti,ab OR inattention:ti,ab OR attention problems:ti,ab OR hyperkinetic disorder:ti,ab OR executive dysfunction:ti,ab  #3 MeSH descriptor: [Child] explode all trees OR MeSH descriptor: [Adolescent] explode all trees OR child*:ti,ab OR adolescen*:ti,ab OR pediatric:ti,ab OR paediatric:ti,ab OR preschool*:ti,ab  #4 #1 AND #2 AND #3 |
| **Web of Science Core Collection** | #1 TS=(screen time OR electronic media OR digital screen OR sedentary screen use OR television OR smartphone OR tablet OR computer use OR digital media)  #2 TS=("attention-deficit/hyperactivity disorder" OR ADHD OR hyperactivity OR inattention OR "attention problems" OR "hyperkinetic disorder" OR "executive dysfunction")  #3 TS=(child OR children OR adolescent OR adolescents OR pediatric OR paediatric OR preschool OR preschooler OR "young children")  #4 #1 AND #2 AND #3 |
| **PsycINFO (via APA PsycNet)** | #1 (DE "Screen Time" OR "screen time" OR "electronic media" OR "digital screen" OR "sedentary screen use" OR "television" OR "smartphone" OR "tablet" OR "computer use" OR "digital media").ti,ab.  #2 (DE "Attention Deficit Hyperactivity Disorder" OR "attention-deficit/hyperactivity disorder" OR ADHD OR hyperactivity OR inattention OR "attention problems" OR "hyperkinetic disorder" OR "executive dysfunction").ti,ab.  #3 (DE "Children" OR DE "Adolescents" OR child* OR adolescen* OR pediatric OR paediatric OR preschool* OR "young children").ti,ab.  #4 #1 AND #2 AND #3 |
